# A Modified Wagner Stem Design Increases the Primary Stability in Cementless Revision Hip Arthroplasty

**DOI:** 10.1016/j.artd.2025.101622

**Published:** 2025-02-03

**Authors:** Julius M. Boettcher, Kay Sellenschloh, Gerd Huber, Benjamin Ondruschka, Michael M. Morlock

**Affiliations:** a Institute of Biomechanics, Hamburg University of Technology, Hamburg, Germany; b Institute of Legal Medicine, University Medical Center Hamburg-Eppendorf, Hamburg, Germany

**Keywords:** Cementless revision hip arthroplasty, Hip revision stem, Primary stability, Implant design, In-vitro experiment

## Abstract

**Background:**

Primary stability is of great importance for the longevity of the implant in cementless revision total hip arthroplasty, since instability is a major cause of rerevision. The purpose of this study was to evaluate the effect of an additional set of less prominent, wider splines added to an established conical stem design with sharp splines on axial stability in a model with significant proximal bone defects.

**Methods:**

Twenty fresh-frozen human femurs were implanted with either the established or the additional spline design, dynamically loaded and tested in a load-to-failure configuration. Cortical contact in the femoral canal after implantation was evaluated by superimposing computed tomography scans and 3-dimensional laser scans. Stem subsidence and micromotion were evaluated to assess primary stability.

**Results:**

Stems remained stable during cyclic loading of up to 200% body weight, except in bones with cortical bone mineral density below 1000 mgHA/mL. A significant reduction of more than 85% in stem subsidence (*P* = .040), axial micromotion (*P* = .007), and rotational micromotion (*P* = .010) was achieved with the new spline design. Load-to-failure testing exceeded 400% body weight.

**Conclusions:**

The new spline design increased the cortical contact which resulted in increased axial primary stability in this in vitro experiment. Bone mineral density as a measure of bone quality proved to be a decisive factor for achieving immediate postoperative stability. Further variations of the established stem designs could further improve the longevity of artificial joint replacements.

## Introduction

Revision total hip arthroplasty (THA) can comprise a significant challenge for the surgeon due to bone defects or poor bone quality in aging patients. Aseptic loosening and overall instability are major causes of hip rerevision, often due to inadequate direct postoperative primary stability resulting in stem migration and rotational motion [1,2]. To counteract this, cementless revision stems must be able to withstand significant loads during daily loading immediately postoperatively [3]. Tapered stems with longitudinal splines based on the Wagner design [4,5] have been shown to withstand these loads and achieve favorable long-term clinical outcomes, even in the presence of significant proximal bone defects compared to alternative treatment options [[6], [7], [8], [9], [10]]. However, the high number of rerevision procedures of about 10% of all primary revisions clearly indicates that initial stability needs to be further improved to allow for bone ingrowth and sufficient implant fixation for favorable long-term survivorship. The German Arthroplasty Registry (Endoprothesenregister Deutschland) documented 18,145 revision THAs compared to 177,826 primary THAs in 2022 [1], while the National Joint Registry for England, Wales, Northern Ireland, the Isle of Man, and Guernsey reported 145,000 revision THAs compared to nearly 1,450,000 primary THAs between 2003 and 2022 [2]. Each revision increases the risk of further revisions [11].

Wagner-type revision hip stems were shown to improve bone regeneration in comparison to alternative design options [12] and stability [13] in a compromised femoral environment, although stem migration has been identified as a concern potentially increasing the risk of instability [14]. Studies have therefore focused on the effect of varying geometry of the distal stem to reduce relative stem motion and migration. The stem design with an additional set of splines investigated in the present study was already shown to exhibit a better resistance to rotational loads [15]. In addition, a study using bone surrogates showed that wider splines and higher taper angles significantly improved axial stability [16].

In light of these findings, the purpose of this study was to evaluate whether the new stem design is superior in terms of micromotion and subsidence if subjected to axial loading in a human bone model.

## Material and methods

Ten pairs of fresh-frozen human femurs (72.3 ± 13.2 years, 5 males, 5 females) were obtained in co-operation with the Institute of Legal Medicine Hamburg [17]. Individual patient data were anonymized. This study was approved by the Ethics Committee of the Hamburg Medical Association (2023-300350-WF).

Computed tomography (CT) scans (Incisive CT 128; Philips, Amsterdam, The Netherlands; voxel size: 0.4 × 0.4 × 0.4 mm³) were acquired with a calibration phantom (QSA; QRM, Moehrendorf, Germany). Hounsfield units were converted to bone mineral density (BMD) with respect to hydroxyapatite content (Structural Insight 3; University Medical Center Schleswig-Holstein [18], Kiel, Germany). Average cortical BMD was determined from a 10-mm wide ring of the femoral shaft at a distance of 5.8% of the body height distal to the minor trochanter (threshold: 400-2000 mgHA/mL [19,20], Matlab R2023a; The Mathworks, Natick, MA). Patient-specific Dorr type and the canal-to-calcar ratio were evaluated as measures of bone morphology following a validated approach by Konow et al. [19]. A spherical region of interest in the center of the femoral head (volume: 1000 mm³) was used to determine trabecular BMD. The position of this region was automatically determined based on a closed-form solution to fit a sphere to the bone above the lesser trochanter by minimizing the algebraic distance between the points and the sphere.

### Proximal bone defects

Cementless long revision stems based on the Wagner design are frequently used for proximal bone deficiencies classified as type IIIA or IIIB according to the classification introduced by Della Valle and Paprosky with little to no proximal bone for proximal fixation [21].

To mimic such a severe, yet frequently occurring revision situation, a defect protocol from a previous study was applied. An extended trochanteric osteotomy (ETO) was performed, which resembles to a procedure that is performed by orthopaedic surgeons to facilitate a controlled primary implant removal [22]. The linea aspera was identified as the posterior landmark for starting the ETO in the distal direction. The incision was made at a length equal to 7.5% of the patient’s height. This corresponds to approximately 120-130 mm. The ETO was then continued from the posterior to the anterior side over 120° using a template and was terminated at the proximal end of the bone [15].

### In-vitro experiment

**Figure 1:**
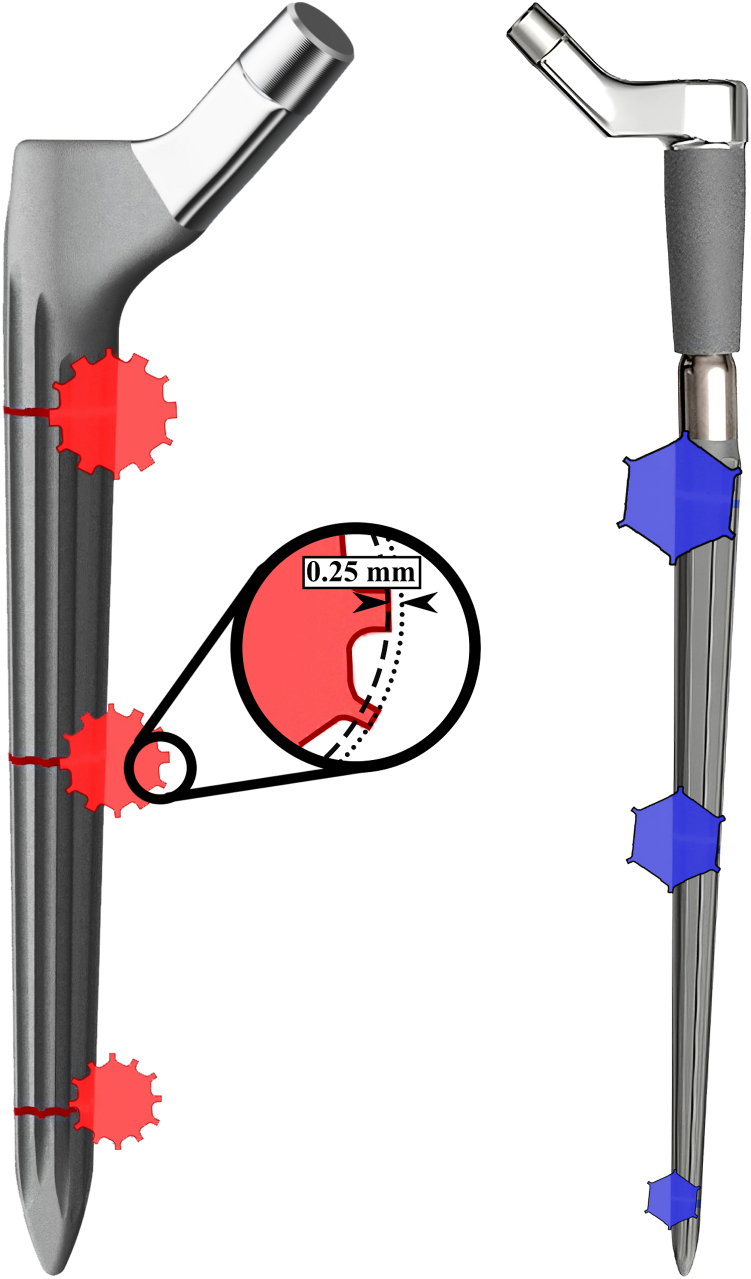
Left: The new RECLAIM Advanced Spline (RAS) design features a second set of wider splines, which are 0.25 mm less prominent than the standard design (right; adapted from [23]).

Revision stems with conventional splines (distal stem, RECLAIM Modular Hip System; Depuy Synthes, Warsaw, IN; stem length: 140 mm) were implanted in 1 bone of each femoral pair. Stems with a new spline design (RECLAIM Monobloc Hip System; Depuy Synthes, Warsaw, IN; stem length: 185 and 235 mm) were implanted on the contralateral side following standard surgical procedures. The new stem design features a second set of less prominent splines compared to the modular stem design called the RECLAIM Advanced Spline (RAS) Design (Fig. 1). Preoperative templating to determine stem size was carried out using the VELYS Hip Navigation software (Version 4.5.0.39; Depuy Synthes, Warsaw, IN).

**Figure 2:**
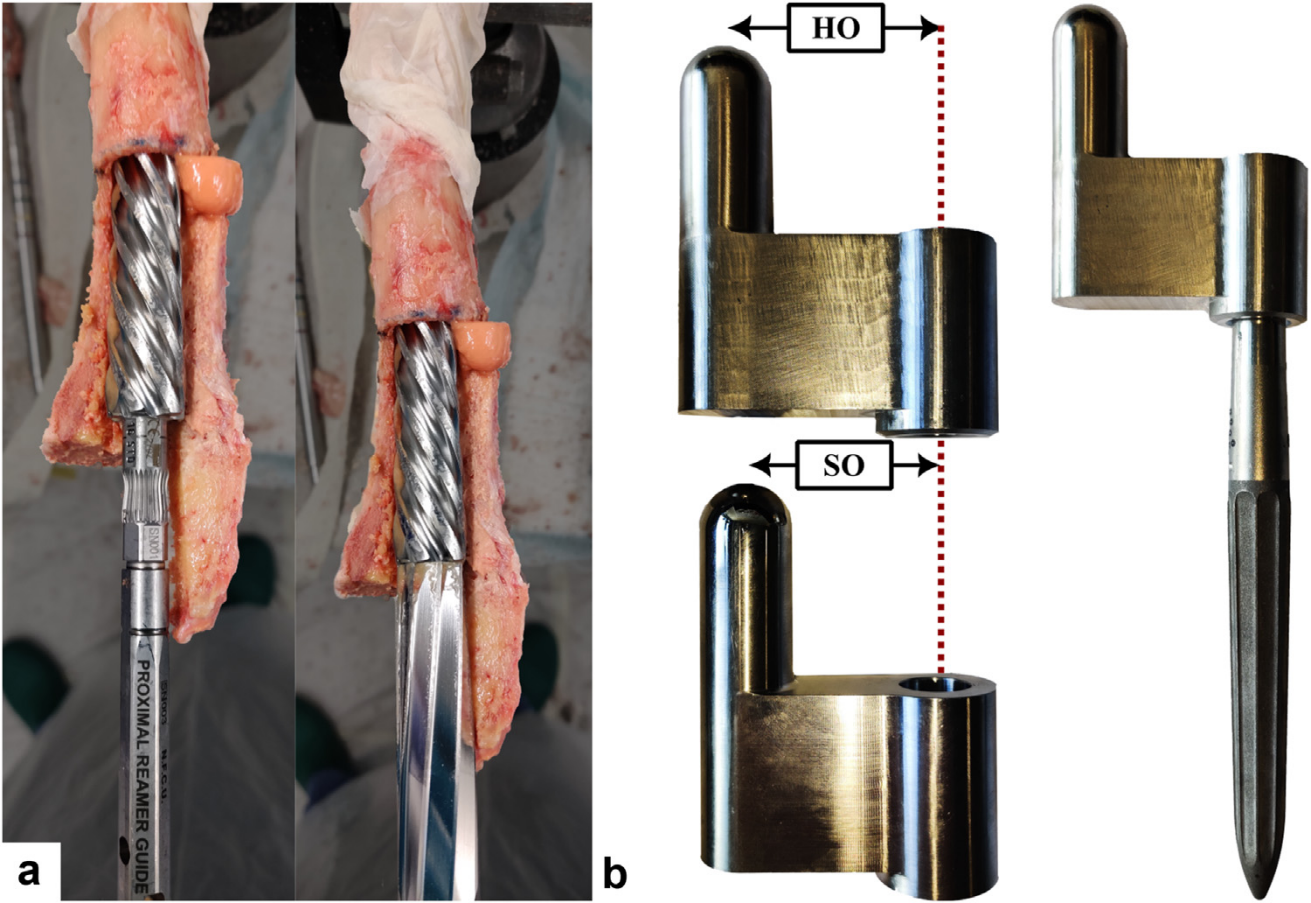
Cavity preparation was performed with a surgical power tool using the appropriate helical reamers according to the respective surgical technique for each implant system after the ETO. (a) Distal and proximal reamers for the monobloc system. (b) Proximal body of the modular stems solely for implant testing. Force application points and offsets (HO: 45 mm, SO: 40 mm) were matched with the respective monobloc stem implanted in the contralateral femur. ETO, extended trochanteric osteotomy.

Powered reaming following the respective surgical technique and to the planned prosthesis size was performed using the respective helical reamers. The colored marks on the instruments were used to determine the final reaming position in relation to the greater trochanter (Fig. 2a). After reaming, the specimens were embedded in metal pods with a 2-component polymer for later fixation on the testing machine (Technovit 4004; Kulzer GmbH, Wehrheim, Germany) and a second femoral CT scan was obtained. Alignment was ensured by positioning the axis of the last reamer perpendicular to a 2-axes spirit level aligned with the femoral axis. The bone samples were wrapped in paper towels soaked in Ringer’s solution, double bagged in sealed plastic bags to prevent freeze drying and then refrozen.

**Figure 3:**
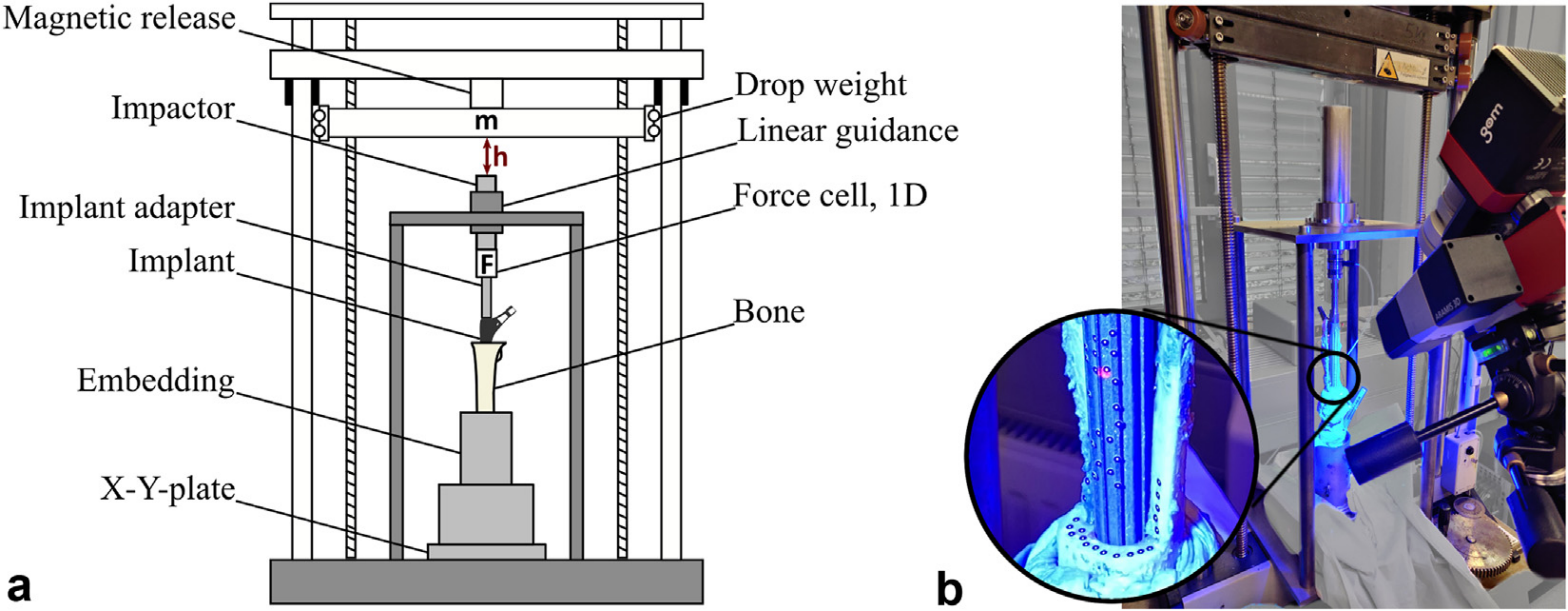
Drop tower setup for controlled stem insertion. (a) Schematic drawing showing the position of the force cell (F) and of the drop weight (m) relative to the implant. Energy levels were defined by the drop height (h). (b) Image of the implantation process with the markers of the DIC system on stem and bone to record the dynamic implant seating. DIC, digital image correlation.

Samples were thawed at 22°C for at least 3 hours on the day of testing. Based on previous studies with isolated human femurs after soft tissue removal, this time frame ensures a fully thawed specimen. The modular distal stems were connected to a proximal body comprised of the original taper junction for the distal stem and similar load application geometries for impaction and loading solely designed for implant testing (Fig. 2b). Force-controlled assembly with a force of 9000 N was achieved using a uniaxial testing machine (0.04 mm/s, Z010, ZwickRoell, Ulm, Germany). Subsequent implantation was performed with a droptower to eliminate variability caused by inconsistent mallet blows (Fig. 3a). The impact energy was increased from 2 J (drop weight: 5 kg; drop height: 40 mm) to 5 J in 1 J increments unless incremental stem seating per hit was less than 0.5 mm. The upper limit equates to a representative blow with a surgical mallet and was set to avoid fractures [24]. Axial impaction forces of each impact were measured between the implant adapter and the impactor using a uniaxial force cell (50 ms, 800 kHz, 9333a; Kistler Instrumente, Winterthur, Switzerland). Dynamic stem seating during implantation was recorded using a digital image correlation system (DIC, Measuring accuracy 0.01 pixel, optimized calibration error analogous to [25], 25 fps, FOV: 2752 × 2200 px, marker size: 0.4 mm; ARAMIS 3-diemsional [3D] Camera; Carl Zeiss, Braunschweig, Germany, Fig. 3b). Stopping criteria were either that the final implant position as reamed was reached or that the implant seating per hit was less than 0.5 mm at 5 J. A seating coefficient τ was evaluated analogous to [15] by fitting exponential functions to the change in seating depth (x) divided by the peak impaction force (F) per hit. A higher seating coefficient was indicative for less effective stem seating.

**Figure 4:**
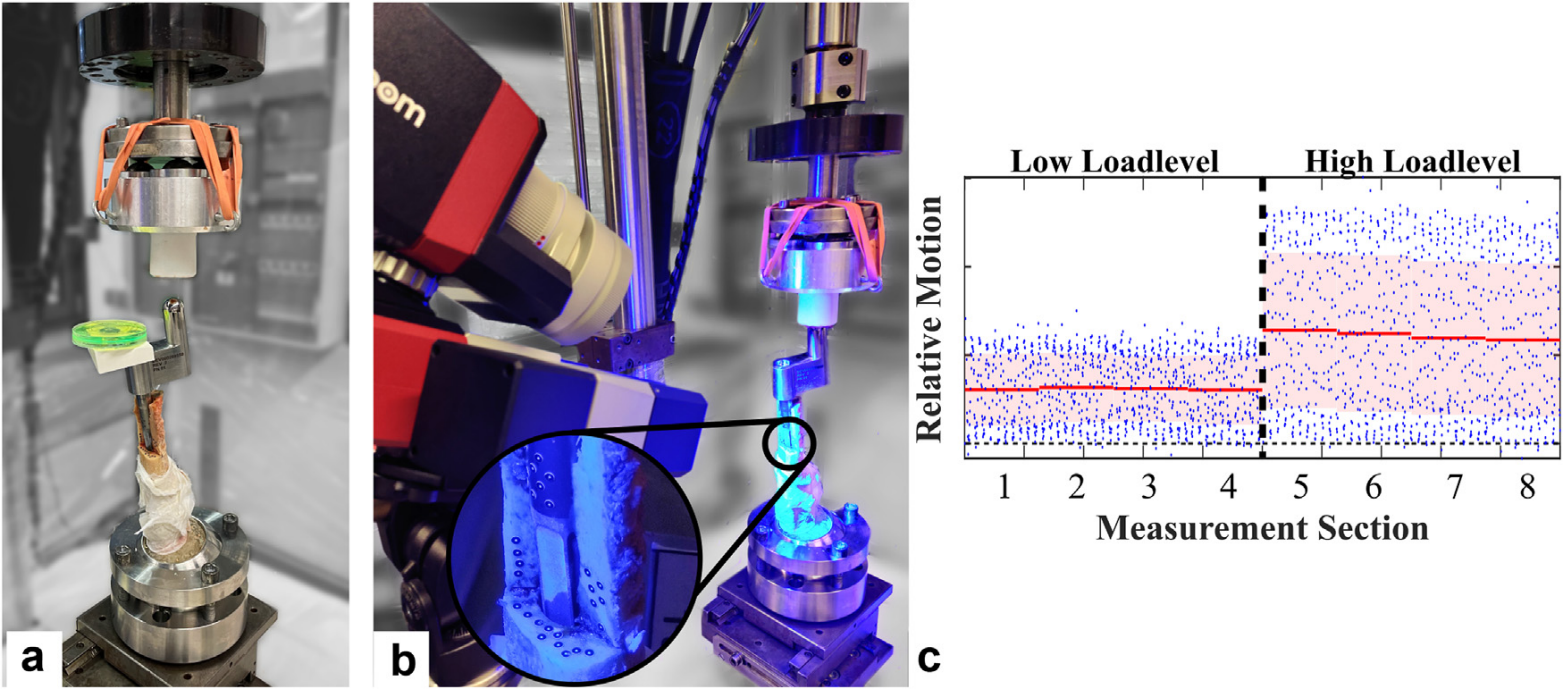
Cyclic loading was applied at 2 load levels using a servo-hydraulic testing machine to determine relative motion between the stem and bone using DIC (example shown for a modular stem). (a) Alignment of the femora according to the ISO standard was ensured using a ball-and-socket clamp and a 2D spirit level together with an adapter. (b) Detail of the optical markers of the DIC system. (c) Relative motion was evaluated in terms of rotation around the implant axis (°) and subsidence (mm) defined by the change of the mean (red) over the different measurement sections. The change in standard deviation (light red) was defined as micromotion at the interface. DIC, digital image correlation; 2D, 2-dimensional.

After implantation, ceramic ball heads (28 mm, 12/14L) were assembled with the monobloc stem tapers. The custom-made proximal body of the modular stems had a surface that was directly suitable as a force application point with identical offsets as the ball head center of the respective monobloc stem implanted in the contralateral femur (standard offset: 40 mm, high offset: 45 mm). The specimens were mounted in a servo-hydraulic testing machine and subjected to cyclic loading (1 Hz) at 2 load levels, each applied for 600 cycles (force-controlled, load level low: peak-to-peak 80-800 N, load level high: peak-to-peak 80-1600 N; MiniBionix II; MTS, Eden Prairie, MN). Alignment according to ISO 7206-4 [26], with a 10° lateral and 9° dorsal tilt of the implant axis with respect to the loading axis, was ensured using a 2-dimensional spirit level and a ball-and-socket clamp as a variable fixture (Fig. 4a). The relative motion between bone and stem was recorded using the DIC system with the same markers as during the implantation measurements every 120 cycles for 10 seconds (Fig. 4b). The relative movement of the implant in the direction of the stem axis and the relative rotation of the implant around the stem axis were determined. The change in the respective mean value of the axial relative movement between the first and last measurement section of the 2 loading steps and between the first and last measurement section of the entire test was defined as the subsidence. The change in the standard deviation of the axial relative motion for the same regions during the same time interval was defined as the micromotion. The standard deviation was chosen for reasons of robustness. For an ideal harmonic oscillation, the standard deviation is approximately 70% of the amplitude. The rotational relative movement was evaluated analogously (Fig. 4c).

After cyclic loading, all specimens were loaded to failure by femoral fracture (displacement-controlled, 0.05 mm/s, MiniBionix II; MTS, Eden Prairie, MN). Force direction and application was similar to the cyclic loading configuration. Load-to-failure was measured with a force cell mounted below the x-y table, and stem subsidence and relative rotation at the ETO position were recorded by the DIC system. The moment of failure of the bone-implant-interface regarding relative movement was defined by a sudden increase of more than 30% of the average calculated from 10 consecutive measurement time points to the neighboring 10 measurements. Resulting moments and force components in the stem coordinate system (positive x-axis medially toward the neck, positive z-axis as the stem axis toward the stem tip) were calculated at the distal end of the ETO and at the stem tip by transforming the force vector in the coordinate system of the load cell to the local coordinate system at the position of the force application. Respective local bending moments were calculated using the lever arms. All bones were kept moist with Ringer’s solution throughout the experiments.

### Contact analysis

**Figure 5:**
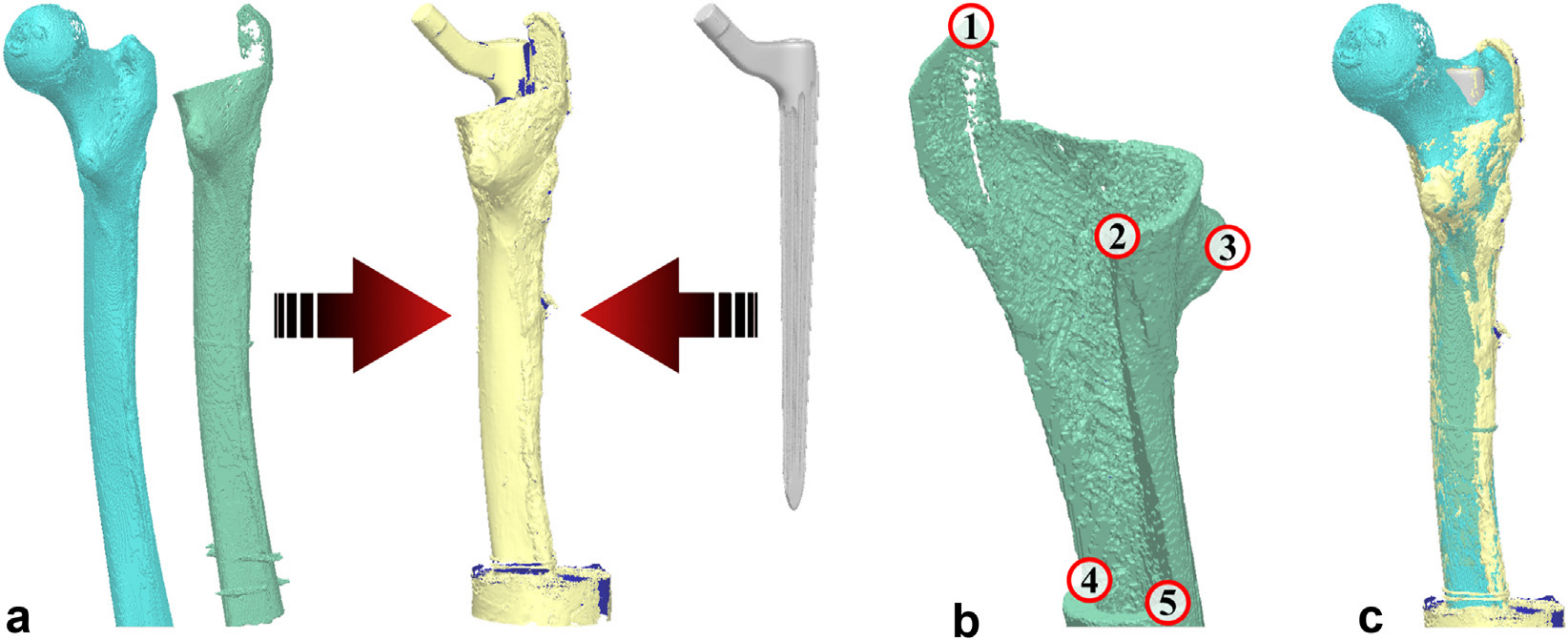
Alignment of the surface models for subsequent contact analysis inside the femoral canal. (a) The 3D laser scan of the implanted stem inside the femur was used as a reference to which the segmented cortices of the native and reamed bone (on the left) and the 3D laser scan of the respective stem (on the right) were aligned using a semi-automatic ICP approach. (b) Five prominent landmarks were manually selected as starting points for the subsequent automatic alignment. (c) Resulting alignment of the surface models. 3D, 3-dimensional; ICP, iterative closest point.

The benefit of the new RAS spline design was investigated by analyzing the contact pattern between stems and cortical bones. Surface models of the cortices were segmented from the CT images (threshold: 400-2000 mgHA/mL, Avizo Lite 2020.2; Thermo Fisher Scientific, Waltham, MA) and superimposed by surface models derived from high-resolution 3D laser scans (scan resolution of 0.012 mm; HandySCAN Black+|Elite; Creaform, Quebec, Canada) of the implants and of the implanted stems together with the bone after implantation. The laser scan of the implant-bone construct enabled alignment of the implant model with the model of the cortical bone with its cavity after reaming or with the segmented native bone (PolyWorks Metrology Suite 2020; InnovMetric Software Inc., Quebec, Canada, Fig. 5a). A semi-automatic iterative closest point algorithm was applied manually selecting prominent landmarks such as the lesser trochanter or the saw-cut incisions of the ETO (Fig. 5b). As optimization criterion for the iterative closest point algorithm, the residual root mean square error was set to below 0.012 mm which corresponds to the resolution of the 3D laser scanner (Fig. 5c).

**Figure 6:**
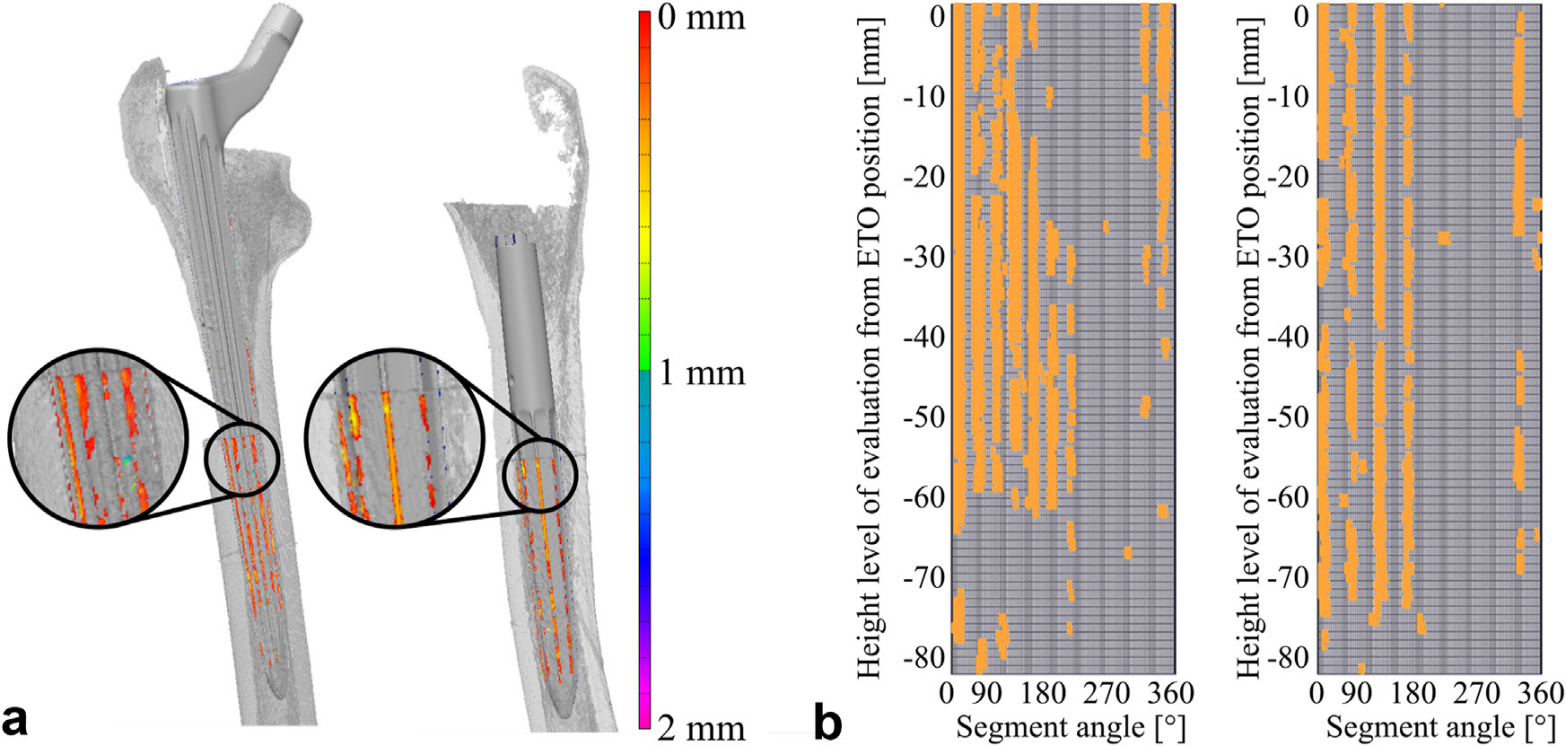
Visualization of contact areas. (a) Alignment of both stem designs in a femur pair. The overclosure and the respective depth of indentation of the splines into the cortical bone are highlighted. (b) An example of the contact matrices showing coherent contact areas for either design (left: RAS, right: conventional). The second set of splines of the RAS design on the left increased the contact compared to the conventional design on the right. RAS, RECLAIM Advanced Spline.

Reaming accuracy was determined based on the angular deviation between the axis of the reamed cavity and the axis of the femoral canal of the native bone evaluated at the same height. The deviation between the templated reaming depth and the actual achieved position of the implant tip after implantation was evaluated in relation to the greater trochanter derived from CT images. The indentation depth of the splines was evaluated as the overlap of the implant models with its splines and the respective reamed bone models in the x and y directions, while the z-axis was defined to coincide with the implant axis. Between the distal saw cut of the ETO and the stem tip, each slice of 1 mm height was divided into 120 sectors of 3° opening angle each, starting at 0° medially. In these sectors, the location and the size of the overlap between the implant and the cortical bone (Fig. 6a) were determined and illustrated as contact matrixes which show coherent contact areas (Fig. 6b).

### Statistical analysis

Statistical analyses were performed with a type I error level of 0.05 (SPSS, version 26; IBM SPSS Statistics, Armonk, NY). Shapiro-Wilk and Levene test statistics were used to test for normal distribution and homoscedasticity. Dependent *t*-tests were used to analyze differences within a pair of femurs. Pearson correlations were used to identify relationships among the parameters analyzed.

## Results

Neither cortical BMD (1103 ± 100 mgHA/mL) nor trabecular BMD (296 ± 92 mgHA/mL) was statistically different between the femur pairs (p_cortical_ = 0.366, p_trabecular_ = 0.228 dependent *t*-tests) and between the 2 groups analyzed (p_cortical_ = 0.879, p_trabecular_ = 0.714 independent *t*-tests). Bone morphology within femoral pairs was similar regarding Dorr type (*P* = .678, dependent *t*-test) and the canal-to-calcar ratio of the femoral canal (*P* = .921, dependent *t*-test).

### Implantation process

Reaming accuracy exhibited an angular difference between the femoral canal and the implant axis for both implant designs with a similar extend for both designs (monobloc: 1.5 ± 0.3°, modular: 1.4 ± 0.7°, *P* = .705, dependent *t*-test). The templated reaming depth was not achieved for either implant design (RAS: 4.6 ± 2.8 mm, conventional: 5.7 ± 2.7 mm, *P* = .488, dependent *t*-test).

The cumulative implantation force required to implant the 2 designs showed no statistically significant difference (RAS: 27.5 ± 17.4 kN. conventional: 30.3 ± 16.1 kN, *P* = .415, dependent *t*-test). Cumulative implantation force was strongly related to cortical BMD (*P* = .001, R^2^ = 0.465, Pearson correlation). No fractures occurred during stem insertion.

**Figure 7:**
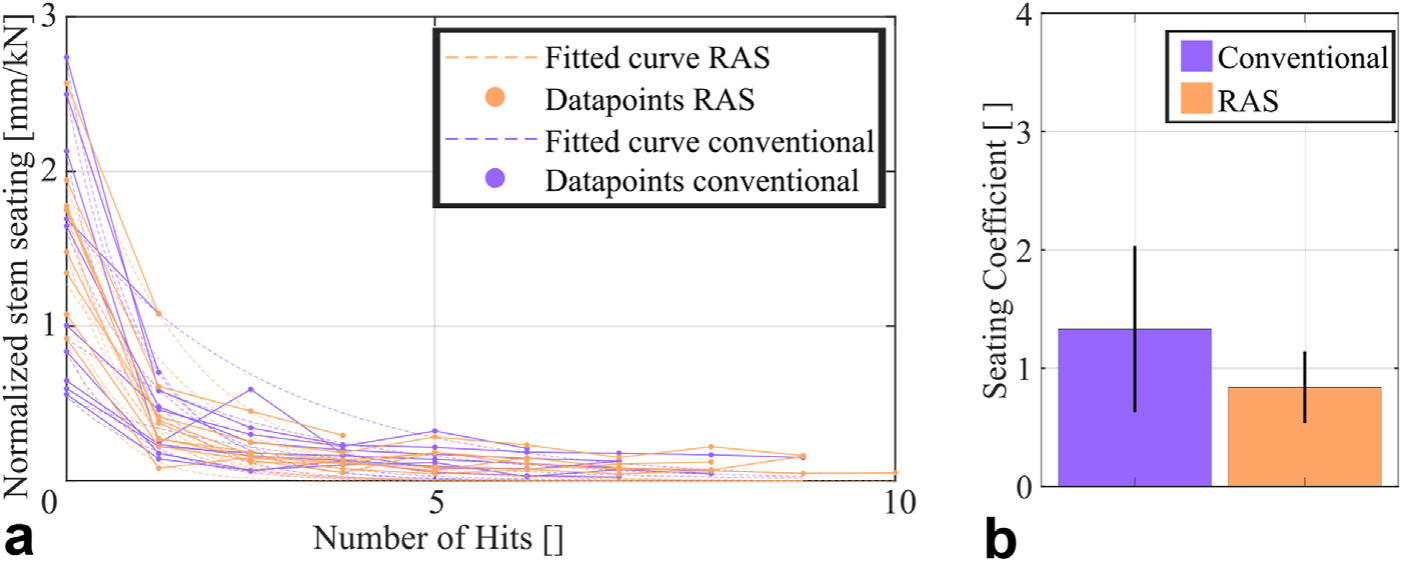
(a) Normalized stem seating was showing a similar course for both designs. (b) A lower seating coefficient indicates faster seating or less force required per mm of seating during implantation. The coefficient was higher for the conventional spline design, but not statistically significant.

The exponential seating coefficient τ was lower for the RAS spline design indicating less force needed to insert the stem but not statistically significant (RAS: 0.84 ± 0.28, conventional: 1.33 ± 0.66, *P* = .057, dependent *t*-test, Fig. 7).

### Cyclic loading

The first load level (80-800 N) exhibited no significant differences between the 2 implant designs during cyclic loading. Both femora from 1 donor fractured within the first 100 cycles. For the second load level (80-1600 N), stem subsidence (RAS: 0.01 ± 0.01 mm, conventional: 0.52 ± 0.64 mm, *P* = .046, dependent *t*-test) and rotational micromotion (RAS: 0.01 ± 0.03°, conventional: 0.1 ± 0.1°, *P* = .047, dependent *t*-test) were significantly reduced for the RAS spline design. Three femur fractures occurred during the second load level. Two of those 3 femurs were again from 1 donor; the third femur fracture occurred with the new RAS spline design. All 5 specimens which failed during cyclic loading were excluded from further analysis.

**Figure 8:**
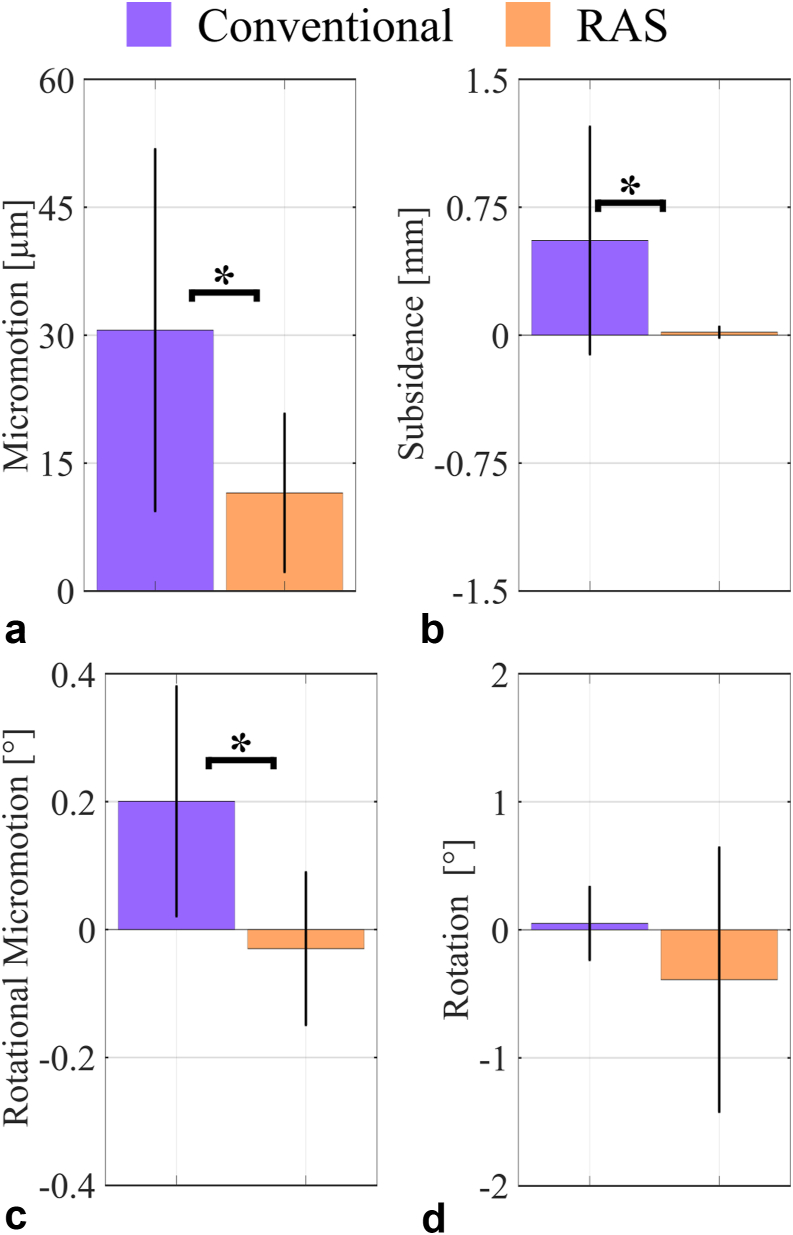
(a) Axial micromotion, (b) subsidence, (c) rotational micromotion, and (d) rotation between the implant and bone due to cyclic loading. Micromotion, subsidence, and rotational micromotion were significantly reduced for the RAS spline design. RAS, RECLAIM Advanced Spline.

The changes in relative motion due to cyclic loading under both load levels were generally low; however, subsidence was significantly reduced by 96.4% (*P* = .040), axial micromotion by 85.2% (*P* = .007), and rotational micromotion by 85.0% (*P* = .010) for the new RAS spline design compared to the conventional spline design (Fig. 8). Absolute subsidence (R^2^ = 0.629) and micromotion (R^2^ = 0.719) were highly dependent on cortical BMD (*P* < .001 for both, Pearson correlations).

### Force to failure

The stems with the conventional spline design failed at 11.6% higher forces compared to the conventional design, which was not statistically significant (RAS: 2.69 ± 0.63 kN, conventional: 3.05 ± 0.55 kN, *P* = .225, dependent *t*-test). The time to failure evaluated by peak force for each specimen during testing was 18.6% more than the time to failure assessed by excessive relative movement between the stem and the femur as recorded by the DIC system (13.3 ± 4.3 s vs 10.8 ± 4.2 seconds, *P* < .001, dependent *t*-test). When comparing the bending moments between the 2 implant designs, no difference was found for the moments at either the ETO position or the implant tip (ETO: *P* = .240, tip: *P* = .368, dependent *t*-tests). No significant correlations were found between bone morphology and density and the evaluated parameters of the force-to-failure test configuration.

### Contact analysis

**Figure 9:**
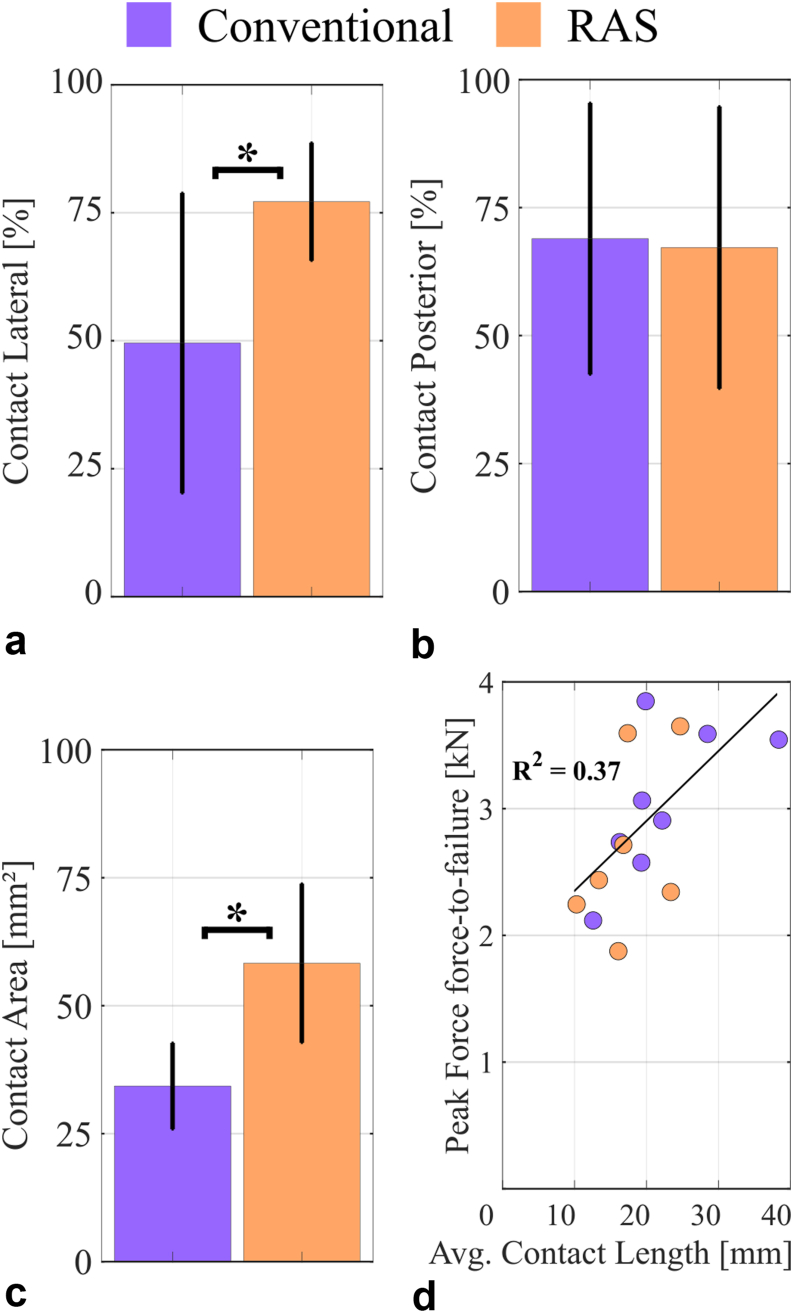
(a) Contact was more lateral than medial for the RAS design. The conventional design showed an even distribution of contact between lateral and medial. (b) Both designs showed an even distribution of contact between posterior and anterior. (c) The cortical contact area was significantly greater for the RAS design compared to the modular stem. (d) Peak force during the force-to-failure test was significantly increased as the average contact length increased. RAS, RECLAIM Advanced Spline.

The RAS spline design with the second set of wider splines achieved a 41.2% increase in contact area compared to the conventional spline design (RAS: 58.3 ± 14.7 mm^2^, conventional: 34.3 ± 8.0 mm^2^, *P* = .001, dependent *t*-test, Fig. 9).

The number of coherent contact areas longer than 5 mm and longer than 10 mm was also significantly increased for the RAS design 30.7 ± 9.6 vs 16.0 ± 4.7, *P* < .001, dependent *t*-test and 23.7 ± 7.7 vs 13.0 ± 4.6, *P* = .001, dependent *t*-test, respectively. The number of coherent contact areas over 30 mm was not significantly different (RAS: 10.8 ± 6.5, conventional: 18.2 ± 9.2, *P* = .140, dependent *t*-test) and showed a positive correlation with the cortical as well as with the trabecular BMD (R^2^_cortical_: 0.228, *P* = .033, R^2^_trabecular_: 0.268, *P* = .019, Pearson correlations).

The average contact length of the contact matrices was not statistically different for the 2 designs (RAS: 14.7 ± 5.1 mm, conventional: 8.3 ± 4.8 mm^2^, *P* = .281, dependent *t*-test). It increased with the cumulative implantation force during implantation (R^2^ = 0.427, *P* = .002, Pearson correlation) and with the peak force during force-to-failure testing (R^2^ = 0.371, *P* = .016, Pearson correlation, Fig. 9).

The RAS design had more lateral than medial contact (77 ± 11%) and more posterior than anterior contact (67 ± 26%), whereas the conventional design exhibited an equal distribution between lateral and medial contact (50 ± 28%). In the ap-direction also, the most contact was found posteriorly for the conventional stem (69 ± 25%). Only the ratio between lateral and medial contact location showed a significant difference between the 2 stem designs (*P* = .029, dependent *t*-test, Fig. 9).

The average indentation depth was similar for the 2 designs (monobloc: 0.19 ± 0.03 mm, modular: 0.19 ± 0.05 mm, *P* = .796, dependent *t*-test) and decreased with cortical BMD (R^2^ = 0.430, *P* = .002, Pearson correlation).

## Discussion

The purpose of this study was to analyze the effect of the new RAS spline design of a cementless revision hip stem on axial stability. It was hypothesized that the addition of a less prominent set of wider splines that engage with the cortex during implantation would increase cortical contact and therefore stabilizes the implant within the bone against axial loading—similar to what had been shown for torsional loading in a foam model [15]. This was confirmed in terms of decreased axial and rotational micromotion and decreased subsidence of the new RAS design compared to the conventional design. The influence of cortical contact on primary stability for cementless implants has already been shown earlier [27,28]. Overall, stem subsidence of either spline design was comparable to values observed in-vitro for the Restoration and Redapt stem design [29] and below radiographic findings reported in the literature [[30], [31], [32]]. Observed micromotions were low and almost 25% below 40 μm, which is regarded as the limit for stable bone ingrowth [[33], [34], [35], [36]]. The observed micromotions are also comparable to previous in-vitro studies on fresh-frozen femora (11.5 μm for the RAS design compared to 13.3 μm of the conventional design [37]).

The lower cumulative implantation force and higher exponential fit coefficient of the RAS design are in contrast to the results of a previous study [15]. This may be explained by the very poor bone quality of the 5 specimens that subsequently failed during cyclic loading. The 2 femurs that became unstable during the first load level only required 2 hits with the lowest energy level during implantation thus reducing the overall average. In addition, slightly smaller angular differences between the native femoral canal and the reamed cavity may have favored a more precise seating during implantation (1.4 ± 0.8° vs 1.6 ± 1.4° in the previous study). Thus, the indentation depth of the primary splines was not increased with the consecutive hits with increased weights, since the wider secondary splines were already in contact with the cortex and prevented further seating. This situation is probably also the reason for the observed deviation between the templated and the achieved implantation depth resulting in a not fully seated implant. Since the templated depth was measured from the cortical bone of the greater trochanter in relation to the reamer length in the CT scan, the final reamer position intraoperatively was most likely biased by residual soft tissue on the greater trochanter, resulting in premature termination of the reaming process. The overall implantation process resulted in a lower seating coefficient for the RAS spline design. This highlights the importance of precise reaming during cavity preparation and the consideration of bone quality for implant selection.

The importance of sufficient cortical BMD was also observed in the cumulative implantation force, subsidence, and micromotion during cyclic loading and illustrated drastically by the 2 femurs with the lowest cortical and trabecular BMD of all specimens, which failed very early during cyclic loading. The 3 specimens which failed during the second load level had the lowest cortical BMD of the remaining specimens. All donors were female, with the oldest being 95 years. Especially with the simulated proximal bone defects, this patient population should clinically rather be treated with a cemented hip revision stem due to insufficient bone quality [38,39].

Peak forces during the force-to-failure tests exceeded peak hip joint forces measured in vivo during daily activities for both implant designs by a factor of 2 despite the prepared proximal bone defects representing a worst-case situation [40,41]. Instability was the first sign of subsequent femoral fracture, as indicated by the significant difference in the total measured time to failure between the applied peak force and the sudden increase in relative movement evaluated by the DIC system. No differences between the 2 spline designs regarding peak forces were found, but the results should be interpreted carefully considering the low statistical power of 0.21, which is a result of the small sample size.

Similar to the previous study investigating the torsional stability of the new RAS spline design [15], contact analysis by aligning and superimposing the stem and bone surface models provided valuable insight into the contact situation between the revision stem and the bone. It was again observed that the less pronounced set of wider splines of the new RAS design get in contact with the cortical shell as intended, but barely indent the cortical bone of the reamed femur. The increased contact area of these splines, combined with the indentation of the thin splines and subsequent circumferential contact over a greater length with sufficient bone quality, results in an increase of the axial primary stability and prevents stem subsidence.

## Conclusions

The area and length of cortical contact between the stem and the femur were found to be a major factor for the immediate postoperative stability of cementless revision hip stems. The new RAS stem design with an additional set of less prominent, wider splines contacting the cortex without indentation, achieved an increase in contact area. This prevents further seating and increases axial stability even in the presence of significant bone defects. Micromotion and peak forces during the force-to-failure test remained below physiological limits. The beneficial effect of additional modified spline designs can potentially be even further increased. Long-term success remains to be demonstrated clinically.

## Conflicts of interest

Gerd Huber received institutional support as a Principal Investigator by DePuy Synthes, Peter Brehm, and Link; is the President of the German Society of Biomechanics. Prof. Dr. med. Benjamin Ondruschka is in the editorial board of “Rechtsmedizin” and “Notaufnahme up2date”; is a board member of the German Society of Legal Medicine. Michael Morlock received speakers bureau/paid presentations for DePuy Synthes, Peter Brehm, and Mathys Enovis; is a paid consultant for DePuy Synthes; and received research support from DePuy Synthes, Ceramtec, and Peter Brehm as a Principal Investigator. All other authors declare no potential conflicts of interest.

For full disclosure statements refer to https://doi.org/10.1016/j.artd.2025.101622.

## Funding

General institutional support was received by Depuy Synthes as indicated in the acknowledgments as well as the provision with surgical components and instruments relevant for this study.

## CRediT authorship contribution statement

**Julius M. Boettcher:** Writing – original draft, Software, Methodology, Investigation, Formal analysis, Data curation, Conceptualization. **Kay Sellenschloh:** Writing – review & editing, Supervision. **Gerd Huber:** Writing – review & editing, Supervision. **Benjamin Ondruschka:** Resources. **Michael M. Morlock:** Writing – review & editing, Supervision, Funding acquisition.

## Appendix ASupplementary data

Conflict of Interest Statement for Ondruschka

Conflict of Interest Statement for Huber

Conflict of Interest Statement for Boettcher

Conflict of Interest Statement for Sellenschloh

Conflict of Interest Statement for Morlock

## References

[1] Grimberg A., Lützner J., Melsheimer O., Morlock M., Steinbrück A. (2023). Endoprothesenregister deutschland (EPRD): jahresbericht 2023.

[2] NJR (2023).

[3] Pilliar R.M., Lee J.M., Maniatopoulos C. (1986). Observations on the effect of movement on bone ingrowth into porous-surfaced implants. Clin Orthop Relat Res.

[4] Wagner H. (1987). Revisionsprothese für das Hüftgelenk bei schwerem Knochenverlust. Orthopä.

[5] Wagner H. (1989). Revisionsprothese für das Hüftgelenk. Orthopä.

[6] Böhm P., Bischel O. (2004). The use of tapered stems for femoral revision surgery. Clin Orthop Relat Res.

[7] Claramunt R.T., Marqués F., León A., Vilà G., Mestre C., Verdié L.P. (2011). Total hip replacement with an uncemented Wagner cone stem for patients with congenital hip dysplasia. Int Orthop.

[8] Hartwig C.H., Böhm P., Czech U., Reize P., Küsswetter W. (1996). The Wagner revision stem in alloarthroplasty of the hip. Arch Orthop Trauma Surg.

[9] Hickie K.L., Neufeld M.E., Howard L.C., Greidanus N.V., Masri B.A., Garbuz D.S. (2024). Long-term outcomes of revision total hip arthroplasty with the Zimmer Modular Revision hip system. Bone Joint J.

[10] Shahin M., Massé V., Belzile É., Bédard L., Angers M., Vendittoli P.-A. (2023). Midterm results of titanium conical Wagner stem with challenging femoral anatomy: survivorship and unique bone remodeling. Orthop Traumatol Surg Res.

[11] Deere K., Whitehouse M.R., Kunutsor S.K., Sayers A., Mason J., Blom A.W. (2022). How long do revised and multiply revised hip replacements last? A retrospective observational study of the National Joint Registry. Lancet Rheumatol.

[12] Del Gutiérrez Alamo J., Garcia-Cimbrelo E., Castellanos V., Gil-Garay E. (2007). Radiographic bone regeneration and clinical outcome with the Wagner SL revision stem: a 5-year to 12-year follow-up study. J Arthroplasty.

[13] Cacciola G., Braconi L., Bosco F., Giustra F., Sabatini L., Capella M. (2023). Outcomes of modular stem for the treatment of periprosthetic femoral fracture: a systematic review of the literature. Ann Jt.

[14] Regis D., Sandri A., Bonetti I., Braggion M., Bartolozzi P. (2011). Femoral revision with the Wagner tapered stem: a ten- to 15-year follow-up study. J Bone Jt Surg Br Vol.

[15] Boettcher J.M., Sellenschloh K., Huber G., Ondruschka B., Morlock M.M. (2023). The influence of hip revision stem spline design on the torsional stability in the presence of major proximal bone defects. PLoS One.

[16] Pierson J.L., Small S.R., Rodriguez J.A., Kang M.N., Glassman A.H. (2015). The effect of taper angle and spline geometry on the initial stability of tapered, splined modular titanium stems. J Arthroplasty.

[17] Püschel K., Heinemann A., Dietz E., Hellwinkel O., Henners D., Fitzek A. (2020). New developments and possibilities in the field of post-mortem medicine mortui vivos docent. Rechtsmedizin.

[18] Graeff C., Timm W., Nickelsen T.N., Farrerons J., Marín F., Barker C. (2007). Monitoring teriparatide-associated changes in vertebral microstructure by high-resolution CT in vivo: results from the EUROFORS study. J Bone Miner Res.

[19] Konow T., Schlieker P.J., Lampe F., Ondruschka B., Morlock M.M., Huber G. (2022). Influence of bone morphology and femur preparation method on the primary stability of hip revision stems. J Orthop Res.

[20] Bätz J., Messer-Hannemann P., Lampe F., Klein A., Püschel K., Morlock M.M. (2019). Effect of cavity preparation and bone mineral density on bone-interface densification and bone-implant contact during press-fit implantation of hip stems. J Orthop Res.

[21] Della Valle C.J., Paprosky W.G. (2003). Classification and an algorithmic approach to the reconstruction of femoral deficiency in revision total hip arthroplasty. J Bone Joint Surg Am.

[22] Wronka K.S., Gerard-Wilson M., Peel E., Rolfson O., Cnudde P.H.J. (2020). Extended trochanteric osteotomy: improving the access and reducing the risk in revision THA. EFORT Open Rev.

[23] DePuy Synthes (2023).

[24] Maharaj G.R., Jamison R.D., Jamison R.D., Gilbertson L.N. (1993). Composite materials for implant applications in the human body: characterization and testing. ASTM International100 barr harbor drive.

[25] Schroeder S., Jaeger S., Schwer J., Seitz A.M., Hamann I., Werner M. (2022). Accuracy measurement of different marker based motion analysis systems for biomechanical applications: a round robin study. PLoS One.

[26] ISO/TC 150 Implants for surgery (2020).

[27] Moriarty P., Sheridan G.A., Wong L., Guerin S., Gul R., Harty J.A. (2020). Bicortical contact predicts subsidence of modular tapered stems in revision total hip arthroplasty. J Arthroplasty.

[28] Meneghini R.M., Hallab N.J., Berger R.A., Jacobs J.J., Paprosky W.G., Rosenberg A.G. (2006). Stem diameter and rotational stability in revision total hip arthroplasty: a biomechanical analysis. J Orthop Surg Res.

[29] Vanhegan I.S., Coathup M.J., McCarthy I., Meswania J., Blunn G.W., Haddad F.S. (2016). An in vitro comparison of the primary stability of 2 tapered fluted femoral stem designs. J Arthroplasty.

[30] Hancock D.S., Sharplin P.K., Larsen P.D., Phillips F.T. (2019). Early radiological and functional outcomes for a cementless press-fit design modular femoral stem revision system. Hip Int.

[31] Park Y.-S., Moon Y.-W., Lim S.-J. (2007). Revision total hip arthroplasty using a fluted and tapered modular distal fixation stem with and without extended trochanteric osteotomy. J Arthroplasty.

[32] Pawar R., Yap R., Blow J., Garabadi M., Rowsell M., Minhas H. (2022). Comparison of two tapered fluted modular titanium (TFMT) stems used in revision hip arthroplasty from a single center. J Orthop.

[33] Engh C.A., O’Connor D., Jasty M., McGovern T.F., Bobyn J.D., Harris W.H. (1992). Quantification of implant micromotion, strain shielding, and bone resorption with porous-coated anatomic medullary locking femoral prostheses. Clin Orthop Relat Res.

[34] Jasty M., Bragdon C., Burke D., Connor D., Lowenstein J.A. (1997). In vivo skeletal responses to porous-surfaced implants subjected to small induced motions. JBJS.

[35] Kohli N., Stoddart J.C., van Arkel R.J. (2021). The limit of tolerable micromotion for implant osseointegration: a systematic review. Sci Rep.

[36] Yang H., Bayoglu R., Clary C.W., Rullkoetter P.J. (2023). Impact of patient, surgical, and implant design factors on predicted tray-bone interface micromotions in cementless total knee arthroplasty. J Orthop Res.

[37] Kirk K.L., Potter B.K., Lehman R.A., Xenos J.S. (2007). Effect of distal stem geometry on interface motion in uncemented revision total hip prostheses. Am J Orthop (Belle Mead NJ).

[38] Della Valle C.J., Paprosky W.G. (2004). The femur in revision total hip arthroplasty evaluation and classification. Clin Orthop Relat Res.

[39] Fink B. (2021).

[40] Huber G., Morlock M.M. (2022). Which length should the neck segment of modular revision stems have?. Clin Biomech.

[41] Bergmann G., Bender A., Dymke J., Duda G., Damm P. (2016). Standardized loads acting in hip implants. PLoS One.

