# Corrigendum to ‘A Modified Wagner Stem Design Increases the Primary Stability in Cementless Revision Hip Arthroplasty’ [Arthroplasty Today 32 (2025) 101622]

**DOI:** 10.1016/j.artd.2025.101657

**Published:** 2025-03-13

**Authors:** Julius M. Boettcher, Kay Sellenschloh, Gerd Huber, Benjamin Ondruschka, Michael M. Morlock

**Affiliations:** a Institute of Biomechanics, Hamburg University of Technology, Hamburg, Germany; b Institute of Legal Medicine, University Medical Center Hamburg-Eppendorf, Hamburg, Germany

The authors regret that the funding statement in the published article is incomplete and should include support from the Funding Programme Open Access Publishing of Hamburg University of Technology.

The full funding statement for this article is confirmed as

## Funding

General institutional support was received by Depuy Synthes as indicated in the acknowledgments as well as the provision with surgical components and instruments relevant for this study. Publishing fees supported by Funding Programme Open Access Publishing of 10.13039/501100023890Hamburg University of Technology (10.13039/501100023890TUHH).

The authors would like to apologise for any inconvenience caused.

