# Supplementary material for: A Modified Wagner Stem Design Increases the Primary Stability in Cementless Revision Hip Arthroplasty
Source: Arthroplast Today. 2025 Feb 3;32:101622. doi: 10.1016/j.artd.2025.101622 (PMC11847092; doi:10.1016/j.artd.2025.101622)
Supplement: Conflict of Interest Statement for Ondruschka [file mmc1.pdf]

# CONFLICT OF INTEREST STATEMENT

## *American Association of Hip and Knee Surgeons*

(Adopted from the American Academy of Orthopaedic Surgeons disclosure statement)

The following form **must be filled out completely and submitted by each author (example, 6 authors, 6 forms). All items require a response. If there is no relevant disclosure for a given item, enter "*None*."**

A modified Wagner stem design increases the primary stability in cementless revision hip arthroplasty

Manuscript Title

1. Royalties from a company or supplier (The following conflicts were disclosed)

*None.*

2. Speakers bureau/paid presentations for a company or supplier (The following conflicts were disclosed)

*None.*

3A. Paid employee for a company or supplier (The following conflicts were disclosed)

*None.*

3B. Paid consultant for a company or supplier (The following conflicts were disclosed)

*None.*

3C. Unpaid consultants for a company or supplier (The following conflicts were disclosed)

*None.*

4. Stock or stock options in a company or supplier (The following conflicts were disclosed)

*None.*

5. Research support from a company or supplier as a Principal Investigator (The following conflicts were disclosed)

*None.*

6. Other financial or material support from a company or supplier (The following conflicts were disclosed)

*None.*

7. Royalties, financial or material support from publishers (The following conflicts were disclosed)

*None.*

8. Medical/Orthopaedic publications editorial/governing board (The following conflicts were disclosed)

*No orthopedic. Editorial board of “Rechtsmedizin” and “Notaufnahme up2date”.*

9. Board member/committee appointments for a society (The following conflicts were disclosed)

*Board member of the German Society of Legal Medicine.*

**Each author must sign AND print or type his/her name, date and submit a separate form**

In addition, one BLINDED Conflict of Interest form (no author names used) should be submitted per manuscript with all author disclosures.

Prof. Dr. med. Benjamin Ondruschka 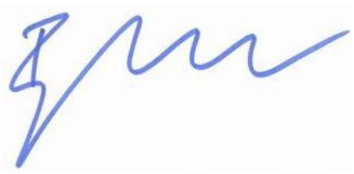

Author Name (Print or Type) | Author Signature | Date 8/7/2024
