# Supplementary material for: A Modified Wagner Stem Design Increases the Primary Stability in Cementless Revision Hip Arthroplasty
Source: Arthroplast Today. 2025 Feb 3;32:101622. doi: 10.1016/j.artd.2025.101622 (PMC11847092; doi:10.1016/j.artd.2025.101622)
Supplement: Conflict of Interest Statement for Morlock [file mmc5.pdf]

# CONFLICT OF INTEREST STATEMENT

## *American Association of Hip and Knee Surgeons*

(Adopted from the American Academy of Orthopaedic Surgeons disclosure statement)

The following form **must be filled out completely and submitted by each author (example, 6 authors, 6 forms). All items require a response. If there is no relevant disclosure for a given item, enter "*None*."**

A modified Wagner stem design increases the primary stability in cementless revision hip arthroplasty

Manuscript Title

1. Royalties from a company or supplier (The following conflicts were disclosed)

   None

2. Speakers bureau/paid presentations for a company or supplier (The following conflicts were disclosed)

   DePuy Synthes, Peter Brehm, Mathys Enovis

3A. Paid employee for a company or supplier (The following conflicts were disclosed)

   None

3B. Paid consultant for a company or supplier (The following conflicts were disclosed)

   DePuy Synthes

3C. Unpaid consultants for a company or supplier (The following conflicts were disclosed)

   None

4. Stock or stock options in a company or supplier (The following conflicts were disclosed)

   None

5. Research support from a company or supplier as a Principal Investigator (The following conflicts were disclosed)

   DePuy Synthes, Ceramtec, Peter Brehm

6. Other financial or material support from a company or supplier (The following conflicts were disclosed)

   None

7. Royalties, financial or material support from publishers (The following conflicts were disclosed)

   None

8. Medical/Orthopaedic publications editorial/governing board (The following conflicts were disclosed)

   None

9. Board member/committee appointments for a society (The following conflicts were disclosed)

   None

**Each author must sign AND print or type his/her name, date and submit a separate form**

In addition, one BLINDED Conflict of Interest form (no author names used) should be submitted per manuscript with all author disclosures.

Michael Morlock

Author Name (Print or Type) | Author Signature | Date 8/8/2024
